# Contribution of GABAa, GABAc and glycine receptors to rat dark-adapted oscillatory potentials in the time and frequency domain

**DOI:** 10.18632/oncotarget.20770

**Published:** 2017-09-08

**Authors:** Jiaman Dai, Juncai He, Gang Wang, Min Wang, Shiying Li, Zheng Qin Yin

**Affiliations:** ^1^ College of Bioengineering, Chongqing University, Chongqing 400030, China; ^2^ Key Lab of Visual Damage and Regeneration & Restoration of Chongqing, Chongqing 400038, China; ^3^ Southwest Hospital/Southwest Eye Hospital, Third Military Medical University, Chongqing 400038, China

**Keywords:** oscillatory potentials, GABA receptors, glycine receptors, frequency domain

## Abstract

Retinal oscillatory potentials (OPs) consist of a series of relatively high-frequency rhythmic wavelets, superimposed onto the ascending phase of the b-wave of the electroretinogram (ERG). However, the origin of OPs is uncertain and methods of measurement of OPs are diverse. In this study, we first isolated OPs from the rat ERG and fitted them with Gabor functions and found that the envelope of the OP contained information about maximum amplitude and time-to-peak to enable satisfactory quantification of the later OPs. And the OP/b-wave ratio should be evaluated to exclude an effect of the b-wave on the OPs. Next, we recorded OPs after intravitreal injection of 2-amino-4-phosphonobutyric acid (APB), tetrodotoxin (TTX), γ-aminobutyric acid (GABA), strychnine (STR), SR95531 (SR), isoguvacine (ISO), (1,2,5,6-tetrahydropyridin-4-yl) methylphosphinic acid (TPMPA) and GABA+TPMPA. We showed that GABA and APB only removed the later OPs, when compared to control eyes. TTX delayed the peak time, and STR, SR and ISO reduced the amplitude of OPs. TPMPA delayed the peak time but increased the ratio of OPs to b-wave. Furthermore, administration of combined GABA and TPMPA caused the later OPs to increase in amplitude with time, compared with those after delivery of GABA alone. Finally, we observed that GABAc and glycine receptors contributed to a low-frequency component of the OPs, while GABAa contributed to both components. These results suggest that the early components of the OPs are mainly generated by the photoreceptors, whilst the later components are mainly regulated by GABAa, GABAc and glycine receptors.

## INTRODUCTION

The electroretinogram (ERG) is a widely used tool for non-invasive measurement of the electrical responses of various cell types in the retina, including the photoreceptors (rods and cones), inner retinal cells (bipolar and amacrine cells), ganglion cells, and Müller cells [1–4]. The ‘a-wave’ is an initial negative deflection in the ERG. It can be divided into two parts, which have different origins: the leading edge of a-wave being produced by hyperpolarized retinal photoreceptors, and the remainder by postsynaptic neurons [5–7]. The ‘b-wave’ (a positive deflection) follows the a-wave and is thought to originate mainly from the activities of ON bipolar cells [4, 8].

The ‘retinal oscillatory potentials’ (OPs) are a series of rhythmic wavelets that appear in the ERG, primarily during the ascending phase of the b-wave [9]. OPs may be useful in the diagnosis of some eye diseases, including diabetic retinopathy [10, 11], retinopathy of prematurity [12, 13], and elevated intraocular pressure [14], amongst others [15]. However, unlike the a- and b-wave, the methods of measurement of OPs are diverse and the origin of OPs is uncertain.

Firstly, the measurement and analysis of OPs is not standardized. Although the extraction of OPs is recommended by the International Society for the Clinical Electrophysiology of Vision (ISCEV) [16], the filter settings used on different equipment can affect the properties of OPs [17, 18]. For quantifying OPs in the time domain, an amplitude index is commonly obtained by summing the amplitudes of the three major peaks [19, 20]. However, others advocate measuring the timing and amplitude of individual peaks [21–24]. Furthermore, it is possible that characterization of OPs dynamics in the frequency domain (using Fourier or wavelet analysis) may reveal additional properties [25–30].

Secondly, there may be some reluctance to use OPs because their physiological origin is relatively poorly understood. It is thought that OPs are mainly generated from negative feedback pathways between bipolar cells, amacrine cells and ganglion cells [9, 31]. A number of studies have indicated that application of γ-aminobutyric acid (GABA) completely removes OPs in both rats and mice [8, 32]. However, there are contradictory reports about the roles of different GABA receptor subtypes in OP generation. Three subtypes of GABA receptor – GABAa, GABAb and GABAc – have been found in the retina [33]. Either antagonism *or* agonism of GABAa can remove OPs [32, 34, 35]. Some studies have shown that GABAb and GABAc receptor blockade has no significant effect on OPs in rats [32], but others report that OPs are increased after blocking GABAc receptors in rats [34, 35] and in GABAc receptor-knockout mice [36].

As mentioned, using frequency domain analysis may offer better insights into the mechanisms of OPs generation. A number of such analyses have demonstrated that two frequency peaks can be seen in the OPs frequency-amplitude spectrum [17, 26]. The origin of these two peaks is uncertain, but they may correspond to two different underlying physiological pathways, for example the rod and cone pathways of the retina [17].

Here, to help clarify these uncertainties, we first studied how best to characterize OPs. We showed that it is reasonable to characterize OPs using the envelope of the OPs, and the ratio of the OPs amplitude to the b-wave amplitude. Next, we demonstrated that OPs decreased in amplitude after blocking GABAa receptors or glycine receptors and increased after blocking GABAc receptors. We also found that GABAc and glycine receptors modulated the low-frequency component of OPs, and GABAa receptors modulated both the low-frequency and high-frequency component of OPs. These results suggested that GABAa, GABAc and glycine receptors contributed differently to the rat dark-adapted OPs in the time and frequency domain.

## RESULTS

### Measurement of the envelope of OPs in the time domain

**Figure 1:**
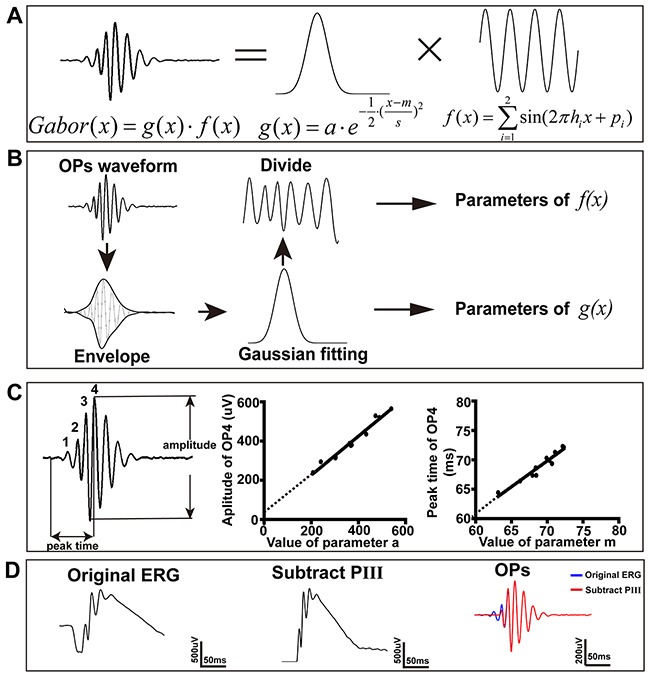
Measurement of OPs in the time domain **(A)** A Gabor function is a Gaussian function, *g*(*x*), multiplied by a sinusoidal carrier function, *f*(*x*). **(B)** Flowchart showing OPs processing. The envelope of the raw OPs was calculated and then fitted with a Gaussian function to get the three parameters of *g*(*x*). The sine wave carrier was then modeled by dividing out the Gaussian envelope and fitting the resulting signal with a double-sine function to get the parameters of *f*(*x*). **(C)** *Left*: Dark-adapted OPs showing the four distinct OPs components (OP1 to OP4). The amplitude and time-to-peak of the largest OPs component (OP4) could be employed as a surrogate for the amplitude and time-to-peak of the overall OPs. *Middle*: Simple linear regression between parameter *a* and the amplitude of OP4. *Right*: Simple linear regression between parameter *m* and the time-to-peak of OP4. **(D)** OP1 and OP2 diminished when subtracting the PIII component from the raw waveforms of ERG.

After the OP was extracted from the ERG waveform, we fitted it with a Gabor function (e.g. Figure 1A) in two stages, as described previously [39] (e.g. Figure 1B). Firstly, we fitted the envelope of the OPs with a Gaussian function (estimating parameters *a*, *m* and *s* of *g*(*x*)), and achieved typical R^2^ values of 0.98 ± 0.013 (n=11). Secondly, after removing this Gaussian component, the remaining sinusoidal carrier was fitted by a two-sine function (estimating parameters *h* and *p* of *f*(*x*)). The reason for selecting a two-sine, rather than single-sine function, was that a single-sine model typically achieved R^2^ values of less than 0.7, whereas a two-sine model typically achieved R^2^ values over 0.95. This is consistent with use in other studies of double-Gabor functions to model OP waveforms [40].

Previous studies have measured the amplitude of OPs as the summed amplitude of each of the wavelet peaks (OP1+OP2+OP3+OP4) [19, 20] or as the individual amplitude of each of the wavelet peaks (OP1, OP2, OP3 and OP4) [21–24]. Here, we propose that it is reasonable to use the amplitude and time-to-peak of the maximum wavelet peak (OP4) as a simple surrogate measure of the properties of the overall OPs. To support this assertion, we showed that both amplitude and time-to-peak of OP4 had a linear relationship with the parameters *a* and *m*, respectively, of the Gaussian model. As shown in Figure 1C, the goodness of fit (as measured by R^2^) of the linear regression between amplitude and *a* was 0.98 (n=11), and between time-to-peak and *m* was 0.99 (n=11). But the width of the function (information of *s)* was missed. Given the close correlation of these parameters (*a* and *m*), and their relative ease of calculation, we suggest that they could be used in a clinical context as simple surrogate indices to represent the properties of the later OPs.

It is reported that the a-wave can complicate the interpretation of the OPs signal, especially for the earlier OP wavelets [13, 41]. To reduce the effect of a-wave on the analysis of OPs, we subtracted the PIII (which includes the a-wave) from the ERG trace, as previously described [41]. As illustrated in Figure 1D, the subtraction of PIII led to the disappearance of OP1 and a reduction in the amplitude of OP2. It is suggested that these earlier OP wavelets may result from the activity of photoreceptors. It can be argued, therefore, that when studying downstream retinal cell activity, it is better to analyze OPs after the removal of the a-wave.

### OP3 and OP4 were generated by the ON pathway

**Figure 2:**
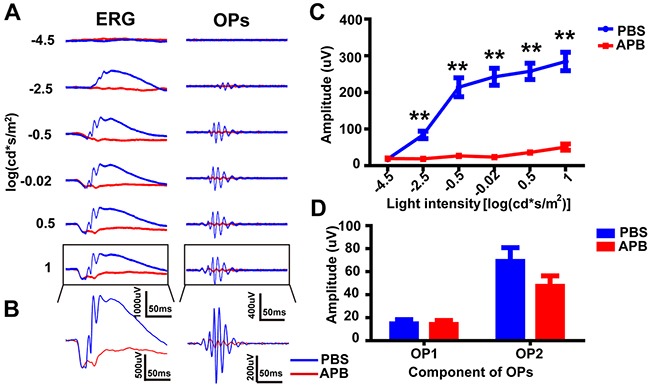
OPs attenuation after intravitreal injection of APB **(A)** *Left panels:* Representative ERG traces evoked by six different light intensities, from −4.5 log(cd·s·m^-2^) at the top to 1 log(cd·s·m^-2^) at the bottom. *Right panels:* OP waveforms isolated from the ERG waveforms by bandpass filtering (60–300 Hz). Red line, APB-treated; blue line, PBS-treated. **(B)** Magnification of those traces outlined in *A*. **(C)** Stimulus-response curves, showing OPs amplitude for APB-treated and PBS-treated eyes (n=6 each). **(D)** Amplitude of OP1 and OP2 in *B* (n=6 each). **p<0.01. Line and bar charts show mean ± SEM.

As introduced above, OPs are typically superimposed onto the ascending phase of the b-wave of the ERG. We asked what would happen to the OPs if the b-wave were also removed. To answer this, we recorded ERGs 10 min after intravitreal injection of 2-amino-4-phosphonobutyric acid (APB, blocker of ON-type bipolar cells), in one eye, and PBS (control), in the contralateral eye (n = 6 for each group). We found that APB completely abolished the ERG b-wave (e.g. Figure 2A, *left panels*. Figure 2B, *left panel* shows an enlargement of the example ERG response to the highest intensity of light). We isolated the OPs using a bandpass filter, as described above, and quantified the amplitude of the OPs response using parameter *a* of the model described previously. Figure 2C shows the intensity-response curves (mean OPs amplitude ± SEM; n = 5) from the APB- and PBS-treated eyes at 10 min post-injection. The OPs amplitude of the APB-injected group was significantly lower than that of the PBS (control) group at light intensities of −2.5, −0.5, −0.02, 0.5 and 1 log(cd·s·m^-2^) (p<0.01 for each by unpaired t-test, two-tailed). Importantly, the main change underlying this fall in OPs amplitude was the dramatic attenuation of the later OP wavelets – OP3 and OP4. In contrast, the earlier OP wavelets (OP1 and OP2) were not significantly reduced in amplitude (Figure 2D, showing result for highest light intensity; APB and PBS not significantly (n.s.) different, by unpaired t-test, two-tailed). These results suggest that the later OP wavelet components were generated directly or indirectly by ON pathway.

### Blockade of glycine receptors reduced OPs amplitude

**Figure 3:**
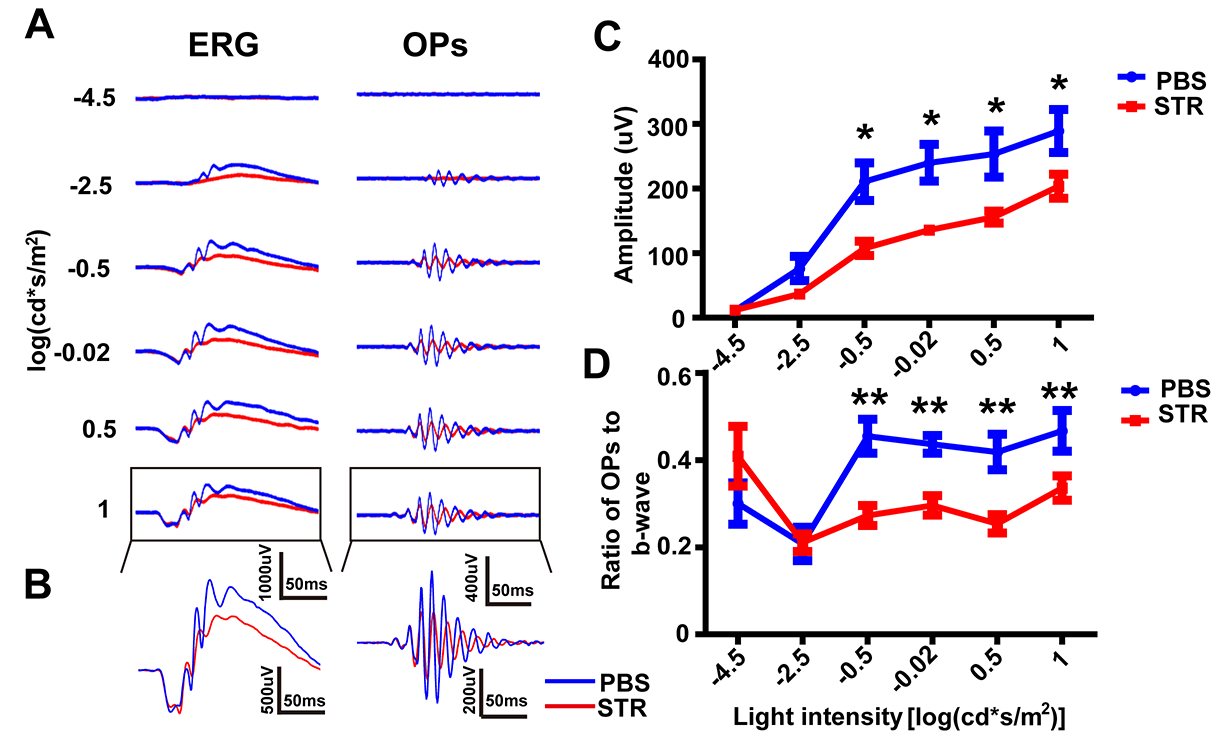
OPs attenuation after intravitreal injection of STR **(A)** *Left panels:* Representative light-evoked ERG traces for different light intensities. *Right panels:* OP waveforms isolated by bandpass filtering. Red line, STR-treated; blue line, PBS-treated. **(B)** Magnification of those traces outlined in *A*. **(C)** Stimulus-response curves, showing OPs amplitude for STR-treated and PBS-treated eyes (n=5 each). **(D)** Amplitude of OP1 and OP2 in *B* (n=5 each). *p<0.05, **p<0.01. Line and bar charts show mean ± SEM.

The signals from rod bipolar cells are directly communicated to glycinergic amacrine cells (AII cells) [42]. To explore the contribution of AII cells to OPs, ERG traces were recorded 10 min after intravitreal injection of strychnine (STR), the antagonist of glycine receptors, or PBS control (contralateral eye) (e.g. Figure 3A, *left panels*). The OP waveforms were then isolated from the ERG (e.g. Figure 3A, *right panels*). The amplitude of OPs in the STR group was significantly reduced compared to the PBS group (Figure 3C; p<0.05 by unpaired t-test, two-tailed). Additionally, the ratio of OP amplitude to b-wave amplitude decreased significantly in the STR-injected group at the four highest light intensities, when compared with those in the PBS-treated group (Figure 3D; p<0.01 by unpaired t-test, two-tailed).

### TTX delayed the peak of the OPs

**Figure 4:**
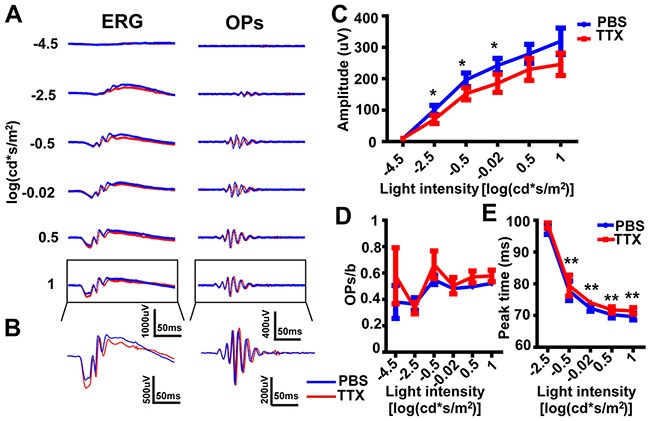
The effect of TTX on dark-adapted OPs **(A)** *Left panels:* Representative light-evoked ERG traces for different light intensities. *Right panels:* OP waveforms isolated by bandpass filtering. Red line, TTX-treated; blue line, PBS-treated. **(B)** Magnification of those traces outlined in *A*. **(C)** Stimulus-response curves, showing OPs amplitude for TTX-treated and PBS-treated eyes (n=5 each). **(D)** Ratio of OPs amplitude to b-wave amplitude at different light intensities. **(E)** Time-to-peak of OPs evoked by the highest five light intensity in *B* (n=5 each). *p<0.05, **p<0.01. Line and bar charts show mean ± SEM.

To evaluate the contribution of amacrine cell and ganglion cell-generated potentials to the OPs, we blocked voltage-gated sodium channels using TTX delivered by intravitreal injection (and PBS into the contralateral eye as a control). ERG traces were recorded 10 min after injection (e.g. Figure 4A) and OP waveforms were isolated (e.g. Figure 4B). The amplitude of OPs in the TTX group was significantly lower at three light intensities (−2.5, −0.5 and −0.02 log(cd·s·m^-2^)), compared with the PBS group (Figure 4C, p<0.05 by unpaired t-test, two-tailed), and the amplitude of the b-wave was also found to be lower in the TTX group (Supplementary Figure 1). Therefore, we observed no significant difference in the OPs/b-wave amplitude ratio between the two groups (Figure 4D, n.s. by unpaired t-test, two-tailed). The most notable effect of TTX (compared to PBS control) was a delay of around 2 ms in the time-to-peak (model parameter “*m*”) of OPs evoked by the last four highest light intensity (Figure 4E, p<0.01 by unpaired t-test, two-tailed). In summary, the main effect of TTX on the OPs was to delay the time-to-peak of OPs.

### Intravitreal injection of GABA abolished the later OPs

**Figure 5:**
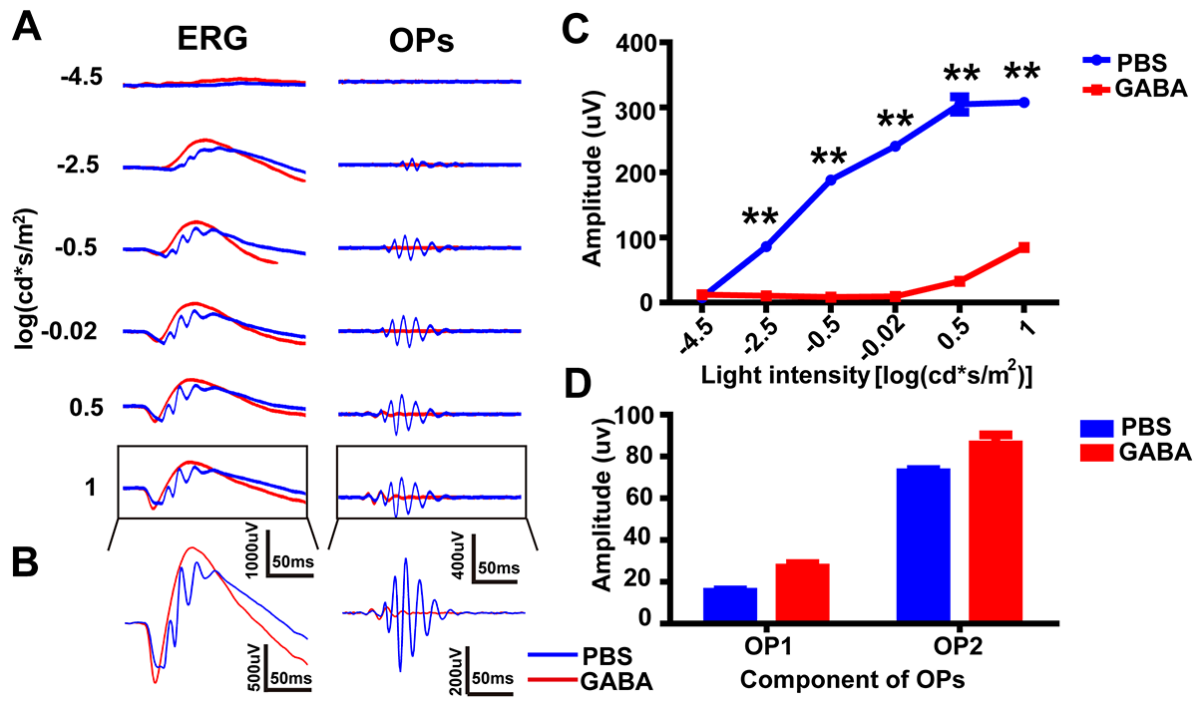
OPs attenuation after intravitreal injection of GABA **(A)** *Left panels:* Representative light-evoked ERG traces for different light intensities. *Right panels:* OP waveforms isolated by bandpass filtering. Red line, GABA-treated; blue line, PBS-treated. **(B)** Magnification of those traces outlined in *A*. **(C)** Stimulus-response curves, showing OPs amplitude for GABA-treated and PBS-treated eyes (n=5 each). **(D)** Average amplitude of OP1 and OP2 in *B* (n=5 each). **p<0.01. Line and bar charts show mean ± SEM.

It has been previously reported that GABA application causes the disappearance of OPs in the eye [8, 32]. To study the effect of GABA on the OPs, we recorded ERGs 10 min after the intravitreal injection of GABA, or PBS control (contralateral eye) (n = 5 for each group, e.g. Figure 5A, *left panels*). OP waveforms were then isolated from the ERG (e.g. Figure 5A, *right panels*). Figure 5B shows magnified versions of the ERGs and isolated OPs for the highest light intensity. Figure 5C shows the intensity-response curves (mean OPs amplitude ± SEM; n = 5) from the GABA- and PBS-treated eyes. At 10 min post-injection, the OPs amplitude of the GABA-treated group was significantly lower than that of the PBS-injected group at light intensities of −2.5, −0.5, −0.02, 0.5 and 1 log(cd·s·m^-2^) (p<0.01 by unpaired t-test, two-tailed). However, this effect was dominated by the attenuation of the later OP wavelets, and the first two OP wavelets (OP1 and OP2) were not significantly smaller following GABA-treatment (Figure 5D; highest intensity shown; n.s. by unpaired t-test, two-tailed). In summary, we found that the intravitreal injection of GABA abolished the later wavelet components of the OPs, but not the earlier wavelet components.

### GABAa and GABAc receptors made differing contributions to later OPs components

**Figure 6:**
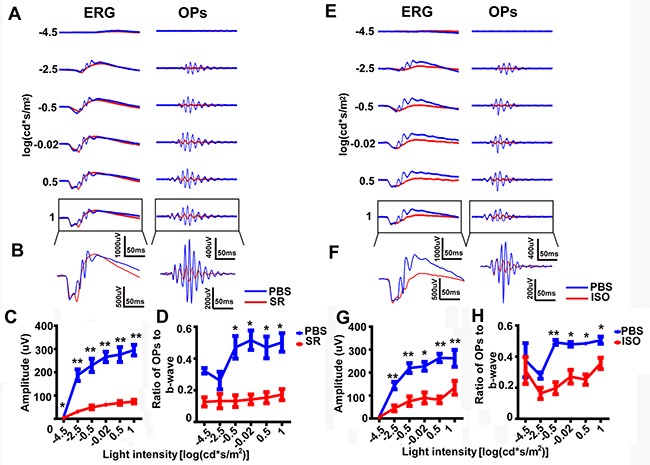
The effect of SR and ISO on dark-adapted OPs **(A)** *Left panels:* Representative light-evoked ERG traces for different light intensities. *Right panels:* OP waveforms isolated by bandpass filtering. Red line, SR-treated; blue line, PBS-treated. **(B)** Magnification of those traces outlined in *A*. **(C)** Stimulus-response curves, showing OPs amplitude for SR-treated and PBS-treated eyes (n=8 each). **(D)** OPs/b-wave amplitude ratio at different light intensities (n=8 each). **(E-H)** As per A–D, but for ISO-treated eyes (red) vs. PBS-treated control eyes (blue). *, p<0.05; **, p<0.01. Line charts show mean ± SEM.

Rod bipolar cells express both GABAa and GABAc receptors [43, 44]. To explore which type of GABA receptor contributes to the OPs, we recorded ERG traces 10 min after the intravitreal injection of SR95531 (SR), a GABAa antagonist, or PBS control (contralateral eye) (n = 8; e.g. Figure 6A, *left panels*). The OP waveforms were then isolated from the ERG (e.g. Figure 6A, *right panels*). As shown in Figure 6A and 6B, the amplitude of the b-wave in the SR-treated eyes increased slightly compared to PBS-treated eyes. However, the amplitude of OPs in the SR group decreased dramatically compared with the PBS group (Figure 6C; p<0.01 by unpaired t-test, two-tailed). Accordingly, the OPs/b-wave ratio was reduced significantly in the SR-treated eyes, compared to PBS-treated eyes, at the four highest light intensities (Figure 6D).

To follow up on this finding, we also recorded ERGs 10 min after the intravitreal injection of the GABAa-receptor agonist isoguvacine (ISO). We found that the amplitude of OPs in the ISO group decreased significantly compared with PBS controls (Figure 6E–6G; p<0.01 by unpaired t-test, two-tailed). Accordingly, the OPs/b-wave ratio reduced significantly in the ISO-treated eyes compared PBS-treated eyes, at the four highest light intensities (Figure 6H, p<0.01, P<0.05 by unpaired t-test, two-tailed).

**Figure 7:**
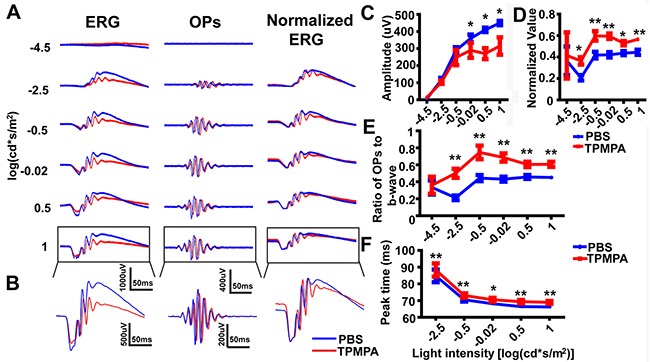
The effect of TPMPA on dark-adapted OPs **(A)** *Left panels:* Representative light-evoked ERG traces for different light intensities. *Middle panels:* OP waveforms isolated by bandpass filtering. *Right panels:* Normalized ERG traces. Red line, TPMPA-treated; blue line, PBS-treated. **(B)** Magnification of those traces outlined in *A*. **(C)** Stimulus-response curves, showing OPs amplitude for TPMPA-treated and PBS-treated eyes (n=5 each). **(D)** Stimulus-response curves, showing normalized OP amplitude for TPMPA-treated and PBS-treated eyes (n=5 each). **(E)** OPs/b-wave amplitude ratio at different light intensities (n=5 each). **(F)** Time-to-peak of OPs evoked by the highest five light intensities (n=5). **p<0.01. Line charts show mean ± SEM.

Next, we tested the effect of TPMPA, an antagonist of GABAc, with ERG traces being recorded 10 min after intravitreal injection (e.g. Figure 7A, *left panels*). The OP waveforms were then isolated from the ERG waveforms (e.g. Figure 7A, *middle panels*). The amplitude of the b-wave in the TPMPA-treated eyes was lower than in PBS-treated eyes. And the amplitude of OPs in the TPMPA group decreased dramatically compared with the PBS group (Figure 7C; p<0.05 by unpaired t-test, two-tailed), at the three highest light intensities. However, the OPs were enhanced in the TPMPA-treated group compared with the PBS-treated group after normalizing the ERG traces (Figure 7A, *right panels.* Figure 7D, p<0.01 by unpaired t-test, two-tailed). And the increased OPs/b-wave ratio confirmed that the amplitude of OPs increased after delivery of TPMPA, compared to PBS control, at the four highest light intensities (Figure 7E, p<0.01 by unpaired t-test, two-tailed). Additionally, the time-to-peak of OPs in the TPMPA-treated eyes was significantly delayed relative to PBS-treated eyes at the five highest light intensities (Figure 7F, p<0.01 by unpaired t-test, two-tailed).

**Figure 8:**
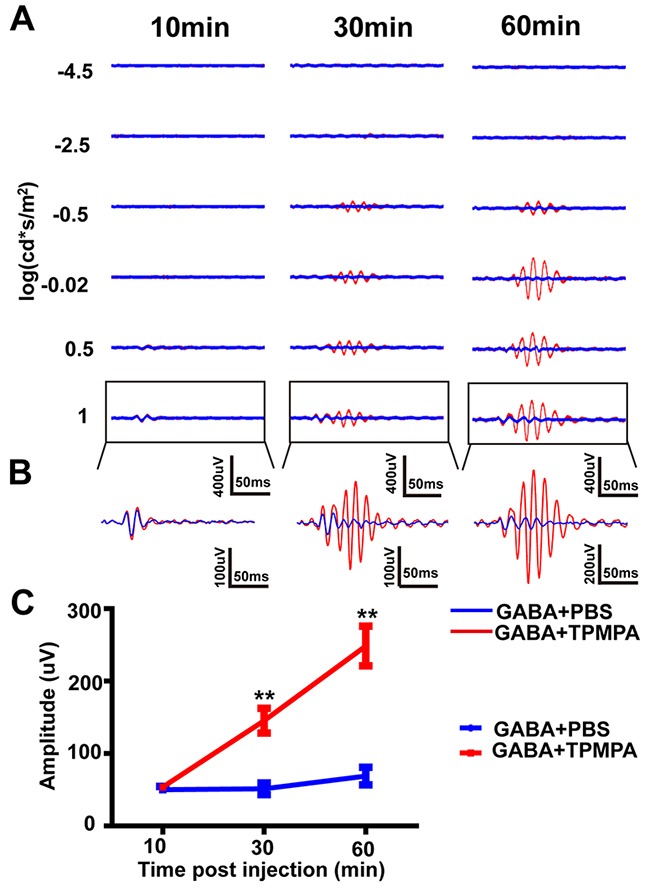
The effect of TPMPA+GABA on the dark-adapted OPs **(A)** Representative OP waveforms, isolated from light-evoked ERG traces, evoked by six different light intensities, and at three different times post-injection. Red line, TPMPA+GABA; blue line, PBS+GABA. **(B)** Magnification of traces outlined in *A*. **(C)** OPs amplitude for TPMPA+GABA-treated and PBS+GABA-treated eyes (n=5) at three different time points after drug delivery. **p<0.01. Line chart shows mean ± SEM.

To further confirm the effect of TPMPA on OPs, a combination of GABA and TPMPA were administered by intravitreal injection. As a control, a combination of GABA and PBS were injected into the contralateral eye. OPs were isolated from ERG waveforms and studied at three time points post-injection: 10, 30 and 60 min (e.g. Figure 8A). At 10 min post-injection, no significant differences in OPs were found between the two experimental groups. At 30 min post-injection, the amplitude of OPs increased significantly in the GABA+TPMPA-treated group compared with the control group (145.43 ± 29.47 μV vs. 57.47 ± 13.90 μV, respectively, at the highest light intensity; p<0.01 by unpaired t-test, two-tailed). One hour post-injection, the difference between the groups increased even further (248.407 ± 27.407 μV vs. 68.890 ± 12.139 μV; p<0.01 by unpaired t-test, two-tailed. These results suggest that TPMPA increased the amplitude of the later OPs.

### GABAa and GABAc made differing contributions to OPs: frequency domain analysis

**Figure 9:**
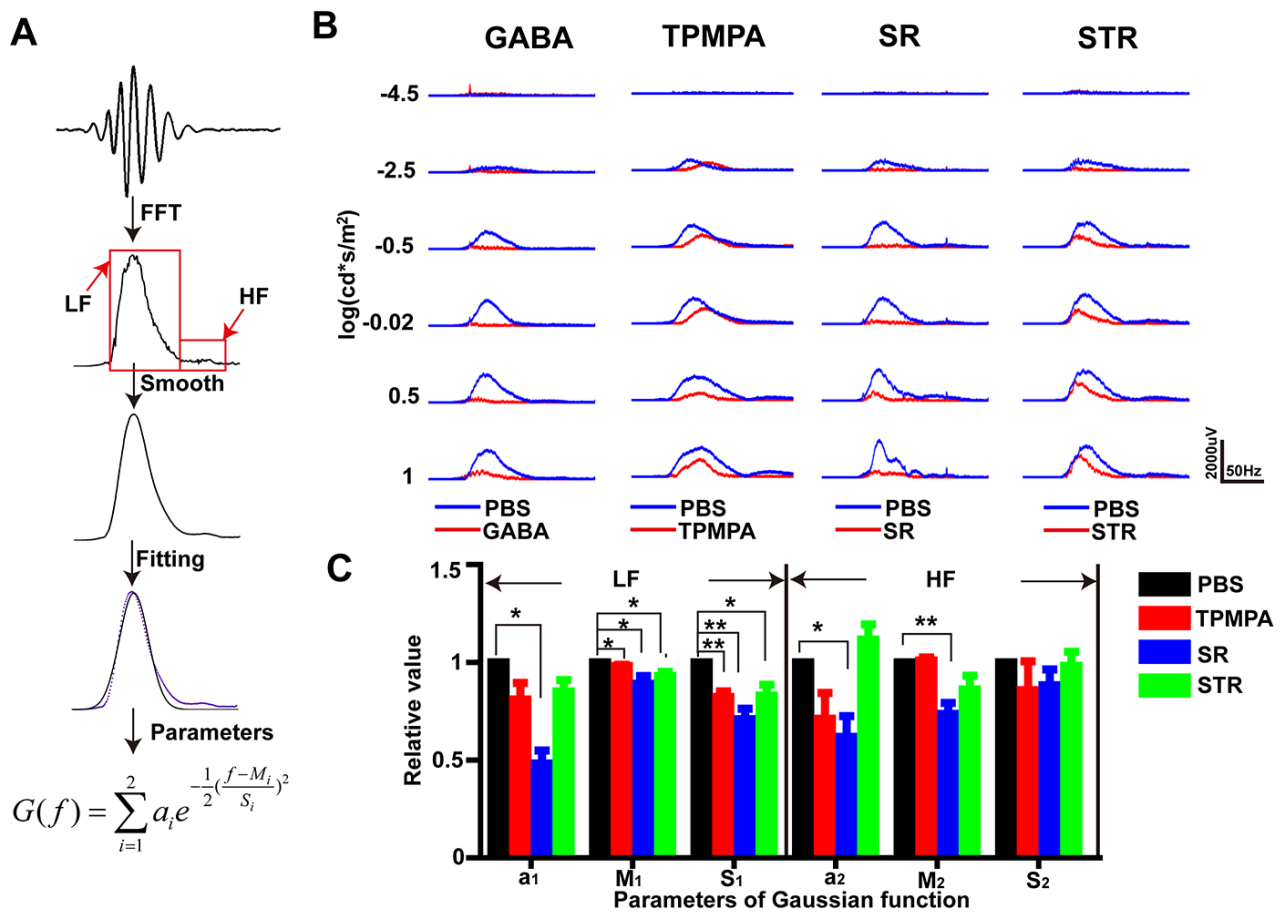
GABAa, GABAc and glycine receptors made differing contributions to OPs: frequency domain analysis **(A)** Flowchart of OPs analysis in the frequency domain. **(B)** Representative frequency-amplitude spectra of the OPs at six different light intensities after administration of different drugs. **(C)** Mean (± SEM) relative values of the parameters of a double-Gaussian model fitted to the data at the highest light intensity (1 log(cd·s·m^-2^)). (n=5, 8, 5). *p<0.05, **p<0.01.

To observe the characteristics of OPs in the frequency domain, Fourier decomposition was performed (using the fast Fourier transform, FFT) on the OPs traces, to reveal the frequency-amplitude spectrum. After viewing the OPs of all the PBS-treated eyes in the frequency domain, we observed that there were two peaks in data: a main, lower-frequency (LF) component, at 75–80 Hz, and a smaller, higher-frequency (HF) component at 130–150 Hz. Next, the frequency-amplitude profile was smoothed, the data were fitted with a double-Gaussian function, and the fitting parameters were used for further analysis. This frequency-domain analysis was performed for six different light intensities and four different experimental treatments (see Figure 9B). The amplitude parameter of the LF component (*a_1_*) in the SR-treated eye was reduced significantly compared with that in the PBS-treated eye (p<0.05, by one sample t-test,). Also, the peak frequency and standard deviation parameters of the LF component (*M_1_* and *S_1_*) in the drug-treated groups TPMPA, SR and STR were all significantly decreased compared to those in the control group (*M_1_*: p<0.05, respectively; *S_1_*: p<0.01, p<0.01 and p<0.05, respectively; by one sample t-test). In the SR-treated group, the amplitude and peak frequency parameters of the HF component (*a_2_* and *M_2_*) significantly decreased when compared with those in the PBS control group (p<0.05, p<0.01, by one sample t-test). These results suggest that GABAc and glycine receptors mainly contributed to the low frequency component while GABAa contributed to both frequency components of OPs.

## DISCUSSION

The aim of this study was to explore the contribution of different types of retinal neurons and neurotransmitters to dark-adapted OPs, in Long-Evans rats. Our results demonstrated several findings that have not been previously reported. Firstly, our analysis suggested that it is reasonable to use parameters from the envelope of the OPs as indices to summarize the properties of the later OPs. Secondly, we showed that the OPs consisted of two main parts: an early part, likely originating from the photoreceptors, and a later part, which could be removed by the application of exogenous GABA or blockade of the ON bipolar cells. Thirdly, GABAa, GABAc and glycine receptor appeared to regulate the later OPs, and contributed differently to the OPs in the frequency domain.

### Measurement of OPs in the time domain

Our findings suggest that it is better to measure the later OPs using parameters that represent the envelope of the OPs, rather than using the properties of the summed OPs wavelet peaks. One reason for this is that these envelope parameters represent the amplitude, time-to-peak and width of the whole OPs. To quantify OPs even more simply, it could be argued that the peak of the largest OPs wavelet (OP4) could be used, since the value of parameter “*a*” in our Gabor-based mode essentially corresponded to the amplitude of OP4, and the value of parameter “*m*” to the time-to-peak of OP4. However, we should acknowledge the limitation that envelope-based methods might not be applicable for analyzing OPs when they are extinguished after drug delivery or in the diseased retina.

Both our mathematical analysis and pharmacological investigation suggest that the two parts of the OPs have different origins. It is important to note that, for the extraction of OPs, the order of the digital filter plays an important role in the outcome [17, 18]. Our pharmacological analysis suggested that the later OPs, but not the early OPs, are abolished by APB (Figure 2). Our results are consistent with previous reports from experiments in the rabbit that the earlier part of OPs originate from the activity of photoreceptors [35]. Because of the different origin of each OP component, we should acknowledge the limitation that the largest component (OP4) will not contain all the information about the OP complex, especially in the diseased retina.

As well as measuring the OP-peaks, we suggest that the ratio of OPs amplitude to b-wave amplitude should also be calculated. In a similar way to how the b/a-wave ratio is already calculated to help identify specific changes in bipolar cells (the gain of the outer retina) [45, 46], the OPs/b-wave ratio could be used to assess the changes in the gain of the inner retina. Our studies show that OPs/b-wave ratio was unchanged in the TTX-treated eye compared that in the PBS-treated eye, although the OPs amplitude decreased with the b-wave (Figure 3). However, after injection of TPMPA, the OPs/b-wave ratio increased, but not the OPs amplitude, when compared with the control group, although the b-wave decreased (Figure 6). Additionally, another approach, normalizing the waveform to the a- and b-wave [47] was used to analyze OPs by eliminating the effect of b-wave. In the TPMPA-treated eye, the increased OPs could be observed from the normalized ERG traces when compared with that in the PBS-treated eye (Figure 7A *right panel*). This suggests that, rather than having no effect on the OPs, TPMPA actually did increase the amplitude of OPs, as has been previously reported [32]. Alongside the suggestion that OPs are removed after abolishing the b-wave by application of APB (Figure 2), these results suggest that the OPs amplitude is affected by the b-wave amplitude. Thus, the ERG OPs/b-wave ratio can be considered an important indicator for evaluating the function of the inner retina especially when measuring the b-wave after removing the OPs.

### Later OPs components are regulated mainly by inhibitory pathways onto rod bipolar cells

Next, we tested the effect of blocking ionotropic glycine receptors by intravitreal injection of strychnine (SR), since AII cells are glycinergic amacrine cells [48]. Both the amplitude of OPs and the OPs/b-wave ratio decreased after administration of strychnine, which is consistent with previous findings that inhibition of glycine transporter type-1 reduced the amplitude of OPs [49]. In the frequency domain, our results suggested that administration of strychnine mainly affected the low frequency component of the OPs (Figure 9C). In summary, we suggest that AII amacrine cells may contribute to OPs by modulating the input to rod bipolar cells via modulation of their glycine receptors (and GABAa receptors). Inhibition of both glycine and GABAa receptors decreased the electrical responses of rod bipolar cells, which is consistent with previous literature [44, 50, 51].

We next examined the contribution of other types of amacrine cells and ganglion cells to the later OPs by intravitreal injection of TTX, a blocker of action potentials generated by amacrine cells and ganglion cells in the retina. The effect of TTX on OPs was to delay the time-to-peak and decrease the amplitude; findings which are consistent with previous reports [52, 53]. Further, it has been suggested that voltage-gated sodium channels contribute to the rodent ERG by regulating input to GABAc receptors on rod bipolar cells [54]. In our results, blocking GABAc receptors delayed the time-to-peak of OPs (Figure 7E). We should emphasize that the OPs in this article were derived from the scotopic ERG, but in the future it will be important to investigate the characteristics of photopic OPs. Together with the results that OPs are unaffected by optic nerve transection [52], we conclude that the contributions of both ganglion cells (and TTX-sensitive amacrine cells) and GABAc receptors to the OPs were to delay the peak time and reduce the amplitude.

Our studies show that application of exogenous GABA removed the later components of OPs, mimicking the effect of APB. However, it has been previously found that GABA completely removes OPs in mice and rats [8, 32]. We speculate that the differences between this study and previous reports may result from the different recording and analysis parameters used, namely the duration of the white-light stimulus (10 μs vs. < 5 μs), the duration of dark-adaptation (45 min vs. >12 h) and the settings of the bandpass filter (100–1000 Hz vs. 50–300 Hz).

We next determined which type of GABA receptors contributed to the later components of the OPs. Here, we showed that inhibiting GABAa by intravitreal injection of SR significantly decreased the amplitude of the later OPs, although it also slightly increased the amplitude of the b-wave. In mice, GABAa-blockade by SR causes complete attenuation of OPs, except for an OP1-like wavelet [34], and it has been reported that SR delays the time-to-peak of the b-wave and increases the amplitude of the b-wave [55]. We suggest that the differences between our b-wave findings and previous reports may result from use of different animal models (rats vs. mice). In the rat, the later components of OPs (in the transretinal ERG) are abolished in the presence of bicuculline [35], which is consistent with our findings.

Interestingly, it has been previously reported that GABAa activation mimics the effect of GABA on OPs [32]. That is to say, either activation or blocking of GABAa receptor led to the later OPs being abolished. We speculate that the effect of activation or blockade of GABAa on the later OPs components results from the wide distribution of GABAa receptors across bipolar cells, amacrine cells and ganglion cells [56]. GABAa may be involved in reciprocal and lateral feedback networks to rod bipolar cells [56] and GABAa may mediate serial inhibition on GABAc expressed on rod bipolar cells [58]. Further studies are needed to explore the exact mechanism of this hypothesis.

We also found that blocking GABAc receptors increased the amplitude of OPs and delayed the time-to-peak of OPs, which is in agreement with previous reports in both rats and mice [34–36]. However, other groups have suggested that GABAc receptors are minimally involved with OPs [32]. These differences in opinion may result from different analysis methods and different recording/processing settings, as described above. To further investigate the contribution of GABAc receptors to the generation of OPs, we injected a combination of TPMAP and GABA. The results suggested that inhibition of GABAc could restore the loss of OPs caused by GABA, which may result from GABAc receptors mediating the majority of lateral GABAergic inputs to rod bipolar cells [50] with GABAc receptors being more sensitive to GABA than the GABAa receptors [43, 57].

Finally, we studied the OPs in the frequency domain, using Fourier analysis. We found evidence of peaks in two frequency bands: a dominant low-frequency (75–80 Hz) component and a smaller high-frequency (130–150 Hz) component, which is consistent with previous reports [17]. Here, we show that GABAa, GABAc and glycine receptors contribute differently to the OPs in the frequency domain. GABAc and glycine receptors contributed to the LF component while GABAa receptors contributed to both frequency components, which agrees with reports that suggest GABAa receptors respond more quickly to GABA than GABAc receptors [50, 58].

In summary, we conclude that: (1) OPs are divided into two parts: an early part and a later part. (2) The origin of these two parts is different. The early components of the OPs seem to be generated by the photoreceptors and the later components are mainly regulated by GABAa, GABAc and glycine receptors, expressed on rod bipolar cells, which may form feedback pathways between rod bipolar cells and amacrine cells. Inhibition of glycine and GABAa receptors decreased the amplitude of the later OPs components, while inhibiting GABAc receptors increased the amplitude of the later OPs components. (3) Frequency domain analysis suggests that GABAc and glycine receptors mainly contribute to a lower-frequency oscillation in the OPs, while GABAa receptors contribute to both lower and higher-frequency components. (4) The envelope of the OPs waveform and the ratio of OPs amplitude to b-wave amplitude may be very useful and practical ways of characterizing OPs in the future.

## MATERIALS AND METHODS

### Rats

All procedures were conducted with the approval of the Third Military Medical University Animal Care and Use Committee. Subjects for this study were Long-Evans rats (postnatal 30 (P30) days, male and female), provided by the Animal Center of the Third Military Medical University, Chongqing, China. The rats were housed in a room with a 12-hour light/dark cycle, with free access to water and food. All the experiments were conducted in accordance with the ARRIVE guidelines [37].

### Intravitreal injection and drug application

All surgical procedures were performed under anesthesia with intraperitoneal injection of ketamine (70 mg/kg) and xylazine (7 mg/kg), and anesthesia was maintained with ketamine (20 mg/kg) and xylazine (1 mg/kg) every 20 minutes. The fundus was examined using direct ophthalmoscopy. As described previously [34], we made solutions of phosphate buffered saline containing 20 mM GABA, 4 mM 2-amino-4-phosphonobutyric acid (APB), 100 μM (1,2,5,6-tetrahydropyridin-4-yl) methylphosphinic acid (TPMPA), 5 μM tetrodotoxin (TTX), 100 μM strychnine (STR), 1mM isoguvacine (ISO) and 100 μM SR95531 (SR). 2 μL of prepared solution was delivered to the vitreous using a Hamilton microsyringe, under dim red light (λ > 620 nm), and the same volume of phosphate-buffered saline (PBS) was injected into the other eye as a vehicle control. The final concentrations of the pharmacological agents used assumed a rat vitreous volume of 50 μL. All drugs were purchased from Sigma-Aldrich (St. Louis, MO, U.S.).

### Electroretinography

ERGs were recorded as described previously [38]. In brief, after 12 hours of dark-adaption, subjects were prepared for recording under dim red-light. Pupils of the anesthetized rats were dilated with one drop of tropicamide and phenylephrine. The recording electrode was a gold wire loop placed on the center of the cornea. A drop of 0.9% saline was frequently applied to prevent corneal dehydration. The reference electrode was a needle electrode inserted under the scleral conjunctiva around the equator of the eyes, and a ground electrode was inserted subcutaneously into the tail. Stimulus presentation, amplification, filtering (0.1–500 Hz band-pass, without notch filtering) and data acquisition were performed by a RETIscan system (Roland Consult, Brandenburg, Germany). All ERG responses presented here are dark-adapted responses, unless specifically mentioned. Stimuli were presented at intensities of −4.5, −2.5, −0.5, −0.02, 0.5 and 1 log(cd·s·m^−2^), and the duration of each stimulus was < 5 us. The sampling rate was 1 kHz. The duration of the pre-stimulus and post-stimulus acquisition period was 150ms and 850ms, respectively. ERG responses were averaged over 3 trials for the weakest two light intensities (and a single trial was used for the higher light intensities). To avoid any adaptation effect of the previous flash, the interstimulus interval was 10–120 s, depending on the flash intensity. The data were output to a computer and processed using MATLAB (v. 2010b, MathWorks, MA, U.S.).

### Offline data analysis

We band-pass filtered the ERG signal offline at 60–300Hz to reveal the OPs (Butterworth, 5^th^ order; mains AC supply frequency in China being 50 Hz). ERG data were then aligned to the onset of the light stimulus, to derive the stimulus-triggered ERG signal. For brevity, the term “ERG” in the remainder of this paper refers to these stimulus-triggered data. The positive peaks of the first four wavelets (which were seen with very high consistency in the ERG) were labeled OP1 to OP4. The amplitude of the largest of these four peaks was documented as the amplitude of the OPs (Figure 1C), and the timing of this maximum OP-peak was calculated relative to the onset of light. The amplitude of other wavelet components, OP1 and OP2, was measured from the prior trough to the peak of that OP component.

Modeling of OPs in the time domain was performed by fitting with a Gabor function, *Gabor*(*x*), as previously described [39]. Briefly, a Gabor function is the multiplication of a Gaussian function with a sinusoidal carrier (e.g. Figure 1A),

$$\begin{array}{cc} \text{Gabor}(x)=g(x)f(x) & \left(1\right) \end{array}$$ (1)

The envelope of the extracted OPs was first calculated by taking the absolute value of the Hilbert transform (provided by MATLAB). Then the envelope was fitted with a Gaussian function, of time, *x*, in ms, relative to light onset,

$$\begin{array}{cc} g(x)=ae^{-\frac{1}{2}{\left(\frac{x-m}{s}\right)}^{2}} & \left(2\right) \end{array}$$ (2)

in which, *a* is the peak amplitude (μV), *m* is the time of the peak (ms) and *s* is the standard deviation (ms) of the Gaussian, which defines the width of the function (Figure 1B). Fitting (here, and below) was achieved with the curve fitting box in MATLAB, using the least square fitting algorithm. This Gaussian component was then removed by division of the OPs signal by the modeled envelope. The remaining signal was fitted with a double sinusoidal function,

$$\begin{array}{cc} f(x)=\sum_{i=1}^{2}\sin(2\pi h_{i}x+p_{i}) & \left(3\right) \end{array}$$ (3)

where *h_i_* is the frequency (Hz) and *p_i_* is the phase (relative to the start of the signal) of the *i*^th^ sinusoid component (*i* = 1, 2).

Analysis of the extracted OPs in the frequency domain was performed as previously described [13]. A fast Fourier transform (FFT) was applied to reveal the frequency-amplitude spectrum of the OPs, which was smoothed (by moving average), then fitted using a double-Gaussian function, *G*(*f*), of frequency (*f*) in Hz,

$$\begin{array}{cc} G(f)=\sum_{i=1}^{2}a_{i}e^{-\frac{1}{2}{\left(\frac{f-M_{i}}{S_{i}}\right)}^{2}} & \left(3\right) \end{array}$$ (4)

where *a_i_* is the maximum amplitude (μV) of the *i*^th^ peak (*i* = 1, 2), *M_i_* is its peak frequency (Hz) and *S_i_* is the standard deviation of the Gaussian kernel (Hz).

For all models, goodness of fit was quantified by the R^2^ value between the original data and the fitted model.

The b-wave was calculated from the trough of the a-wave to the peak of the b-wave. And the OPs/b-wave ratio was evaluated by the amplitude of OPs (amplitude of OP4) dividing the amplitude of b-wave. Normalized ERG traces (unity-based normalization) were obtain by equation

$$\begin{array}{cc} E_{N}=\frac{E_{i}-E_{\min}}{E_{\max}-E_{\min}} & \left(5\right) \end{array}$$ (5)

where *E_max_* is the maximum value of the whole ERG dataset (μV), *E_min_* is the minimum value of the whole ERG dataset (μV), *E_i_* is data of ERG (μV) and *E_N_* is the normalized data of ERG.

Relative values of the parameters from Equation 4 were obtained using the equation

$$\begin{array}{cc} P_{R}=\frac{P_{e}}{P_{c}} & \left(6\right) \end{array}$$ (6)

where *P_R_* is the relative value, *P_e_* is the value of the parameter from the experimental eye and *P_c_* is the value of the parameter from the control eye.

### Statistics

All data are presented as the mean and standard error of the mean (mean ± SEM). The data were evaluated using unpaired/one sample *t*-tests, to compare the means at the same time points between the experimental and control groups. Values of p < 0.05 were considered significant.

## SUPPLEMENTARY MATERIALS FIGURE



## Abbreviations

APB: 2-amino-4-phosphonobutyric acid
ERG: electroretinogram
FFT: fast Fourier transform
GABA: γ-aminobutyric acid
ISO: isoguvacine
OPs: oscillatory potentials
SR: SR95531
STR: strychnine
TPMPA: (1,2,5,6-tetrahydropyridin-4-yl) methylphosphinic acid
TTX: tetrodotoxin
