# Supplementary material for: Contribution of GABAa, GABAc and glycine receptors to rat dark-adapted oscillatory potentials in the time and frequency domain
Source: Oncotarget. 2017 Sep 8;8(44):77696–709. doi: 10.18632/oncotarget.20770 (PMC5652335; doi:10.18632/oncotarget.20770)
Supplement: Supplementary file 1 [file oncotarget-08-77696-s001.pdf]

Contribution of GABAa, GABAc and glycine receptors to rat dark-adapted oscillatory potentials in the time and frequency domain

SUPPLEMENTARY MATERIALS

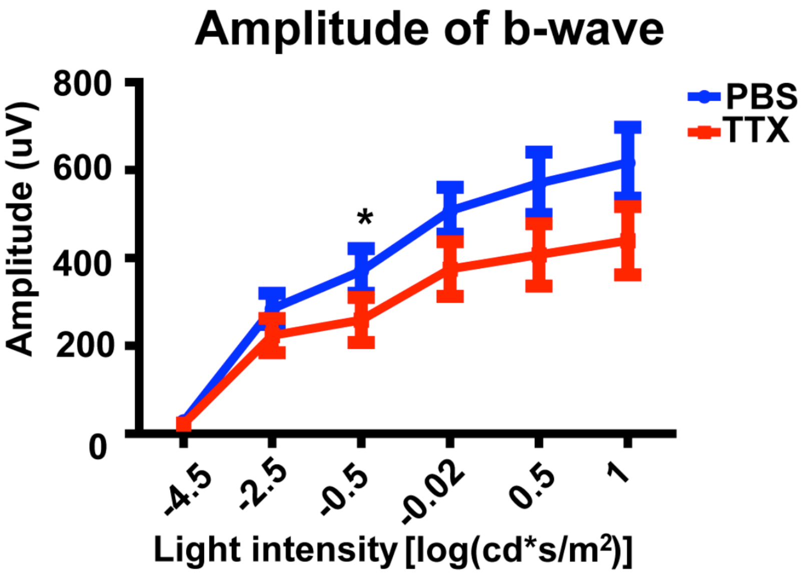

Supplementary Figure 1: Stimulus-response curves, showing b-wave amplitude for TTX-treated and PBS-treated eyes (n=5 each). *$p<0.05$.
